# Transcriptome sequencing reveals high isoform diversity in the ant *Formica exsecta*

**DOI:** 10.7717/peerj.3998

**Published:** 2017-11-21

**Authors:** Kishor Dhaygude, Kalevi Trontti, Jenni Paviala, Claire Morandin, Christopher Wheat, Liselotte Sundström, Heikki Helanterä

**Affiliations:** 1 Centre of Excellence in Biological Interactions, Department of Biosciences, University of Helsinki, Helsinki, Finland; 2 Department of Biosciences, Neurogenomics Laboratory, University of Helsinki, Helsinki, Finland; 3 Department of Zoology Ecology, Stockholm University, Stockholm, Sweden; 4 Tvärminne Zoological Station, University of Helsinki, Hanko, Finland

**Keywords:** Pool-seq, RNA-sequencing, Ants, Transcriptome *de novo* assembly, Hymenoptera, Transcriptomics

## Abstract

Transcriptome resources for social insects have the potential to provide new insight into polyphenism, i.e., how divergent phenotypes arise from the same genome. Here we present a transcriptome based on paired-end RNA sequencing data for the ant *Formica exsecta* (Formicidae, Hymenoptera). The RNA sequencing libraries were constructed from samples of several life stages of both sexes and female castes of queens and workers, in order to maximize representation of expressed genes. We first compare the performance of common assembly and scaffolding software (Trinity, Velvet-Oases, and SOAPdenovo-trans), in producing *de novo* assemblies. Second, we annotate the resulting expressed contigs to the currently published genomes of ants, and other insects, including the honeybee, to filter genes that have annotation evidence of being true genes. Our pipeline resulted in a final assembly of altogether 39,262 mRNA transcripts, with an average coverage of >300X, belonging to 17,496 unique genes with annotation in the related ant species. From these genes, 536 genes were unique to one caste or sex only, highlighting the importance of comprehensive sampling. Our final assembly also showed expression of several splice variants in 6,975 genes, and we show that accounting for splice variants affects the outcome of downstream analyses such as gene ontologies. Our transcriptome provides an outstanding resource for future genetic studies on *F. exsecta* and other ant species, and the presented transcriptome assembly can be adapted to any non-model species that has genomic resources available from a related taxon.

## Introduction

Phenotypic variation can arise via differences in gene sequences or patterns of gene expression (Carroll, 2008; Simpson, Sword & Lo, 2011). Adaptive polyphenism is one of the most dramatic examples of how variation in gene expression can be translated into alternative phenotypes, the castes of social Hymenoptera (ants, social bees and social wasps) being a prime example (Gräff et al., 2007; Bonasio et al., 2010; Hunt et al., 2011; Colgan et al., 2011). The behavioural, physiological, and morphological differentiation between workers and reproductive queens, underlie the adaptive radiations and ecological importance of social Hymenoptera, especially ants (Wilson & Hölldobler, 2005). A diploid, fertilized hymenopteran egg is totipotent, i.e., has the genetic prospects to develop into a queen or a worker, and the developmental trajectory the egg takes among these alternatives is, with a few exceptions (Helms Cahan & Keller, 2003; Pearcy et al., 2004; Fournier et al., 2005), directly influenced by the nutrition and rearing conditions provided by workers (Schwander et al., 2010). These conditions presumably launch a cascade of differential gene expression of a few genes, with large pleiotropic effects at early larval instars causing them to develop into different female castes (Lattorff & Moritz, 2013). Importantly, caste determination is also controlled by epigenetic mechanisms that are regulated by the social environment, e.g., workers (Gräff et al., 2007; Weil, Korb & Rehli, 2009; Glastad et al., 2011; Zwier et al., 2012; Simola et al., 2013; Simola et al., 2016; Welch & Lister, 2014; Bonasio, 2014; Alvarado et al., 2015; Ashby et al., 2016).

Genetic causes and consequences of caste polyphenism have been studied mostly using preselected candidate genes (Abouheif, 2002; Hunt & Goodisman, 2010; Shbailat, Khila & Abouheif, 2010; Feldmeyer, Elsner & Foitzik, 2014), or based on phylogenetically limited comparisons of distantly related taxa (Hunt & Goodisman, 2010; Hunt et al., 2010; Ometto et al., 2011; Berens, Hunt & Toth, 2015). Current genomic resources of social insects are available for important pollinators such as honey bees and bumble bees (Colgan et al., 2011; Kocher et al., 2013; Elsik et al., 2014; Elsik et al., 2016; Kapheim et al., 2015; Sadd et al., 2015), and primitively eusocial paper wasps (Sumner, Pereboom & Jordan, 2006; Ferreira et al., 2013). For ants, genome sequences are currently available for 13, and transcriptomes for 23, species in the Fourmidable database (Wurm et al., 2009), including well studied species such as the invasive argentine ant *(Linepithema humile)*, the fire ant *(Solenopsis invicta),* and the leaf cutting ant species *(Acromyrmex echinatior* and *Atta cephalotes* (Bonasio et al., 2010; Suen et al., 2011; Smith et al., 2011a; Smith et al., 2011b; Wurm et al., 2011; Nygaard et al., 2011; Gadau et al., 2012; Oxley et al., 2014; Schrader et al., 2014; Konorov et al., 2017). However, these data cover only a fraction of the genetic richness of the more than 11,000 described ant species, and over 100 million years of evolution (Wilson & Hölldobler, 2005). More genomic resources are therefore required for phylogenetically informative comparisons that thoroughly assess the genomic consequences of sociality.

RNA-sequencing (high throughput sequencing of transcriptomes) is a relatively inexpensive way to rapidly sequence the coding and expressed genes of a species (Vera et al., 2008). However, there are technical challenges in using the resulting transcriptome assembly (TA) data from non-model organisms in downstream applications in a robust manner, posing problems for the kind of comparative work outlined above. First, *de novo* transcriptome libraries usually have contaminations with exogenous RNA from e.g., the microbial flora of the species (Bonfert et al., 2013), as demonstrated by a meta-transcriptome of our study species (Johansson et al., 2013). Second, TA’s include transcripts that fail to be annotated, and align poorly to even moderately related species. Such unannotated contigs (contiguous consensus sequences that are derived from collections of overlapping reads in the assembly process) may include orphan genes, or variable, and less known regulatory non-coding RNA (Wissler et al., 2013). However, unannotated contigs may also arise from assembly errors, intron retention, or non-functional transcripts, such as pseudogenes (Graveley et al., 2011). As long as the true status of these remains unsolved, removing them from the TA increases its accuracy, and facilitates species comparisons, yet this process also leads to loss of information. Third, unique loci in the genome may be represented in a TA by dozens of predicted isoforms, which typically are not assembled completely. Given this, and the potential for overprediction of isoforms (i.e., false isoforms), care must be taken to properly use the TA to obtain unbiased read counts for each gene (Tulin et al., 2013). One approach is to identify likely gene-isoform relationships, and sum the reads mapping to each of these fragments and isoforms, that are inferred to come from the same locus (Hornett & Wheat, 2012).

Here we present a transcriptome of the ant *Formica exsecta* (Formicidae; Hymenoptera), a common species throughout the Palearctic region. It has morphologically well-separated queen and worker castes, and forms perennial nests of some thousands of workers (Brown, Keller & Sundström, 2002; Vitikainen, Haag-Liautard & Sundström, 2011; Vitikainen, Haag-Liautard & Sundström, 2015). Genetic and social nest structure varies greatly, and each nest can have one or several queens that have mated with one or many males, which makes it a model species for kin conflicts (Sundström, Chapuisat & Keller, 1996; Brown, Liautard & Keller, 2003; Kummerli & Keller, 2007), social recognition (Martin et al., 2008; Martin et al., 2009; Van Zweden et al., 2011), and causes and consequences of inbreeding (Sundström, Keller & Chapuisat, 2003; Haag-Liautard et al., 2009; Vitikainen, Haag-Liautard & Sundström, 2011; Vitikainen, Haag-Liautard & Sundström, 2015).

We characterise the transcriptome of *Formica exsecta,* including different sexes, life stages, and castes to obtain a good representation of expressed genes with their possible isoforms, potentially useful as a genomic resource for future studies on ants, as well as other insects. The transcriptome was generated from paired-end RNA-seq on seven libraries: cocoons of workers and queens, young workers and queens newly emerged from cocoons, overwintered adult workers and queens, and a pooled library of males (cocoons and young males newly emerged from cocoons). In order to avoid the TA-related challenges outlined above, we took additional steps of documenting biological evidence for the discovered contigs, by validating them with published ant genomes and genomes of other insects, and clustering the predicted isoforms to unique genes. We also show that different life stages/castes of ants can have different genes expressed. This provides more comprehensive information on the potential number of expressed genes, and their isoform numbers in ants. Furthermore, we provide a commented guideline on the comparison of analysis procedures, i.e., how to obtain overall expression profiling without a reference genome.

## Methods

### Sampling and library sequencing

Samples were collected from six localities within a range of 50 km on the Hanko Peninsula, and the islands outside Tvärminne Zoological Station, SW Finland, between late April and July 2011 (Bioproject: PRJNA213662, Biosample: SAMN02297446–SAMN02297452 Johansson et al., 2013; Morandin et al., 2014; Morandin et al., 2015). Old reproductive queens and old overwintered workers were sampled directly from field colonies in late April and early May, when the queens aggregate at the colony surface. Old males are not available for analyses as they do not live past the mating season. Queen, male, and worker cocoons were collected from the same populations in late June and early July the same year, at an age when sex and caste can be visually assessed. The cocoons consisted of three stages: young (white cuticle and eyes), intermediate (white cuticle but pigmented eyes), and old (pigmented cuticle and eyes). Young males and queens were sampled prior to mating when they appeared at the surface of the nest, and were about to leave for their nuptial flight. Young workers were sampled within a week of eclosion, while still pale, compared to old workers. All samples were frozen directly after collections, and placed in −80 °C awaiting RNA extraction.

The samples were cleaned from visible exogenous material under a preparation microscope, and the total RNA was extracted from whole-ground ants individually in TriSure (Bioline, London, UK), following the manufacturers protocol. RNA quality was determined by assessing the integrity of ribosomal RNA with BioAnalyzer total RNA kit (Agilent, Santa Clara, CA, USA), and denaturing agarose electrophoresis. Subsequently each sample was pooled into seven RNA libraries (Table 1), so that total RNA of each sample had equal representation in the pool. The exception to this was the library of old workers, which could be equalized only between locations, due to the low quality of many extractions derived from old workers. The read data, and the details on RNA pools and sampling locations used here, correspond to the BGI-sequenced libraries in Johansson et al. (2013). In short, altogether 105 individuals from 56 colonies were included in the libraries, and seven RNA libraries were constructed to cover sexes, female castes, and life stages; the paired-end sequencing libraries were constructed and sequenced by the Beijing Genomic Institute BGI (China), according to the provider’s pipeline. The total RNA pools were DNAse-treated and selected for mRNA using poly-A-tail selection, and subsequently fragmented. Approximately 200 base insert length fragments were selected for library construction. Paired-end sequencing was conducted on Illumina HighSeq 2000 platform, 90 bases paired-end reads at Beijing Genomics Institute (BGI Illumina, Inc.). The raw reads of the transcriptome are available on GenBank (https://www.ncbi.nlm.nih.gov/sra/) under Bioproject ID PRJNA213662, SRA accession numbers: SRR945908, SRR945909, SRR945910, SRR945911, SRR945912, SRR945913, and SRR945914.

**Table 1:** RNASeq sequencing libraries. Caste, age, and the number of individuals pooled in each of the 7 libraries sequenced by BGI.

Caste	Age	# Individuals	# Colonies	Sequencing library
Queen	Old overwintered	4	4	Library 1- Old Queen
	New	8	4	Library 2- New Queen
	Old cocoon	6	3	Library 3- Queen Cocoon
	Intermediate cocoon	6	3	
	Young cocoon	6	3	
Worker	Old overwintered	30	8	Library 4- Old Worker
	New	15	6	Library 5- New Worker
	Old cocoon	6	3	Library 6- Worker Cocoon
	Intermediate cocoon	6	3	
	Young cocoon	6	3	
Male	Adult	3	3	Library 7- Male Mix
	Old cocoon	3	3	
	Intermediate cocoon	3	3	
	Young cocoon	3	3	

### Transcriptome assemblies

**Figure 1:**
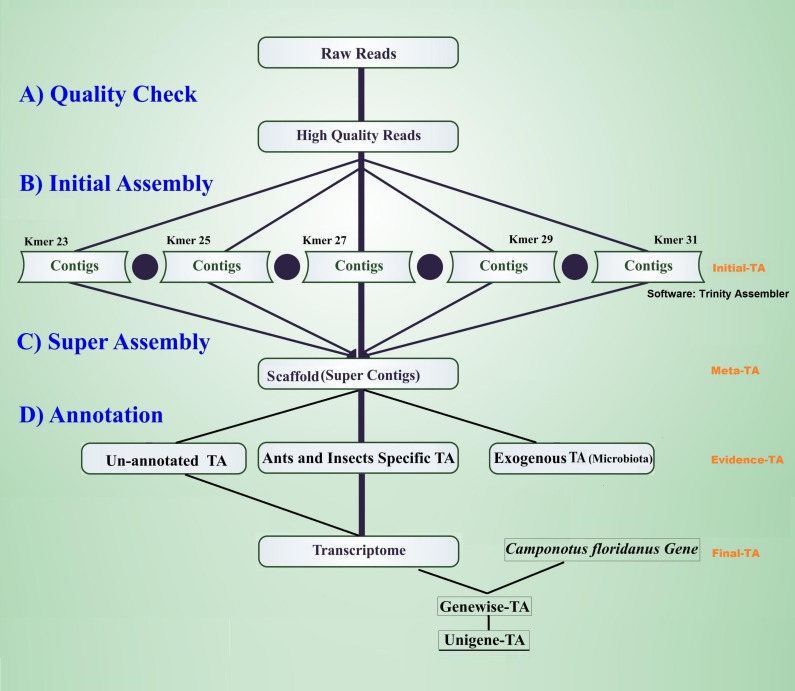
Workflow for the transcriptome assembly. High quality reads were assembled in contigs using five different k-mer settings (initial assembly), and merged (super assembly). The resulting contigs were investigated for evidence of being true species genes. False isoforms due to SNP variation, and sequencing errors were removed by self-alignment, and the remaining contigs with isoforms were assigned to genes using alignment to proteins of related species.

The workflow of the assembly process is outlined in Fig. 1. Before the assembly process, we combined multiple raw data files generated from different sexes, life stages, and castes (Table 1). Altogether the combined raw data contained RNA expression profiling data from 105 individuals. To assess the quality of each sequencing library, quality checks were performed separately without combining raw data. The quality of raw reads was assessed separately for forward and reverse reads with FastQC (Andrews, 2010). Forward and reverse reads were trimmed with the FastX toolkit (Version 0.0.13 Hannon, 2010) to equal length of 76 bp by removing the last 14 bp at the 3′-ends. After trimming, all reads were free from Illumina adapter contamination and primer dimers, paired data were without any orphan reads, and were of high quality (average ≥ 20 Q for all base positions on Phred scale). This resulted in 322 million clean read pairs for the TA (raw read processing summary table in File S1, Table S1A). These trimmed reads were assembled in four different stages of TA (Initial-TA, Meta-TA, Evidence-TA, and Unigene-TA) (Fig. 1).

####  Initial-TA assembly

In order to increase our confidence in the quality of the data, the initial *de novo* transcriptome assemblies (Initial-TA) were carried out using three commonly used transcriptome assembly software: Trinity (release 2012-05-18 (Grabherr et al., 2011; Haas et al., 2013), Velvet-Oases (version 0.2.06 (Schulz et al., 2012)), and SOAPdenovo-trans (version 2011-11-12 (Xie et al., 2014)). Each assembler was run with a range of k-mer values (Trinity k-mers: 21 to 31, Velvet-Oases k-mers: 51 to 75 and SOAPdenovo-trans k-mers: 21 to 71 File S1, Tables S1B–S1D), within the applicable range of the software. The minimum accepted contig length was set to 100. Of the three assemblers, we selected as the best TA assembler the method that covered the greatest proportion of orthologous genes with high coverage (>90% alignment coverage) in the genome of *Camponotus floridanus*. These are applicable not only to the assessment of transcriptome assemblies, but also annotated gene sets. For this comparison, we first constructed a blast database of *Camponotus floridanus* predicted proteins, CflorPP90, containing 15,977 proteins after removing redundant sequences from the original sets (17,064 proteins). Redundant sequences were removed by clustering *Camponotus floridanus* proteins, such that each cluster of similar sequences, with at least 90% sequence identity and 90% overlap, were represented by the longest sequence. This was done using in-house scripts (Wheat, 2017). Second, we aligned the Initial-TA contigs of each assembler (different k-mer settings) to the predicted protein set CflorPP90 using BLASTx (ncbi-blast-2.2.2), with the minimum requirement of 50% amino acid identity, and an alignment length of at least 50 amino acids. Finally, we also recorded the number and length distributions of contigs arising from each assembly method (average contig length and N50), to compare the behaviour of different assemblers at different settings, but these values were not used as an assembly quality proxy (Hornett & Wheat, 2012; O’Neil & Emrich, 2013). We also made separate Trinity assemblies for each caste (male, queen, worker; Table 1.) with the default k-mer, and defined them as Male-TA, Queen-TA and Worker-TA. These caste specific assemblies were used in further evaluations of the final transcriptome assembly (Final-TA) by running BUSCO (Simão et al., 2015) assessment with Hymenoptera lineage specific orthologous gene sets.

#### Meta-TA assembly

The next assembly version (Meta-TA) was produced by combining the contigs that were obtained from all k-mer runs of the Initial-TAs from the Trinity assembler. This was done in order to assemble as many transcripts as possible, as short k-mer values are typically more sensitive than longer ones in detecting rare transcripts. Hence, short k-mers tend to produce more transcripts than long k-mers, whereas long k-mers typically produce fewer, but longer, intact contigs (Surget-Groba & Montoya-Burgos, 2010). We used Vmatch (Abouelhoda, Kurtz & Ohlebusch, 2002) to generate non-redundant sets of transcripts by combining contigs from different k-mer specific trinity assemblies, using default parameters, and by setting the minimum overlap for two contigs to collapse to be at least 100 bases, with a sequence identity of at least 95%. During scaffolding, contigs with very small differences were combined into a consensus sequence. This unavoidably removes some true variation, but we assume most of these isomorphs are artefacts, resulting from SNP and indel variation in the RNA of the pooled individuals, and from sequencing and assembly errors (Nakamura et al., 2011).

#### Evidence-TA assembly

To select TA contigs with evidence of being ant or other insect genes, we compared the Meta-TA contigs against non-redundant (NR) protein datasets from the NCBI database (Updated 2015), and swissprot databases using Blast2Go (BLASTx, *e*-value threshold 10^−3^ (Conesa et al., 2005)). This blast outcome was used to retrieve annotation, gene ontology terms (GO), enzyme annotation, and information of the protein family. Contigs annotated with known micro-organisms, and probable parasites such as viruses and fungi, were first filtered out from the data as exogenous material, as described in Johansson et al. (2013), and this exogenous material has now been made available in NCBI database (Bioproject: PRJNA412141). The remaining contigs were then aligned (BLASTx, *e*-value threshold 10^−3^) to (a) the predicted gene sets of published ant genomes available on the Fourmidable database, including the closest species such as *Camponotus floridanus*, *Lasius niger,* as well as more distantly related species such as *Cerapachys biroi*, *Linepithema humile*, *Solenopsis invicta*, *Vollenhovia emeryi*, *Wasmannia auropunctata*, *Pogonomyrmex barbatus*, *Monomorium pharaonis*, *Harpegnathos saltator*, *Acromyrmex echinatior* and *Atta cephalotes* (Wurm et al., 2011; Wurm et al., 2009; Bonasio et al., 2010; Suen et al., 2011; Smith et al., 2011a; Smith et al., 2011b; Nygaard et al., 2011; Gadau et al., 2012; Oxley et al., 2014; Schrader et al., 2014; Konorov et al., 2017), (b) the honey bee genome (Weinstock et al., 2006), (c) the predicted gene sets of *Nasonia vitripennis*, *Tribolium castaneum* (Wurm et al., 2009), and *Drosophila melanogaster* (Gramates et al., 2017), (d) as well as non-redundant (NR) protein datasets available in the NCBI database (Updated 2015, Updated 2017) for insect species. A minimum requirement of 70% amino acid identity, and at least 100 bases (33 amino acids) alignment length was used. The *L. niger* genome has been added with the NCBI 2017 database update. Contigs finding a match in these data sets were considered as true expressed genes, and form the TA supported by biological evidence (Evidence-TA assembly). This approach is conservative, as choosing only genes supported by annotation evidence may exclude genes with very high divergent sequence evolution, and hence possibly some orphan genes (Tautz & Domazet-Lošo, 2011).

#### Unigene-TA assembly

**Figure 2:**
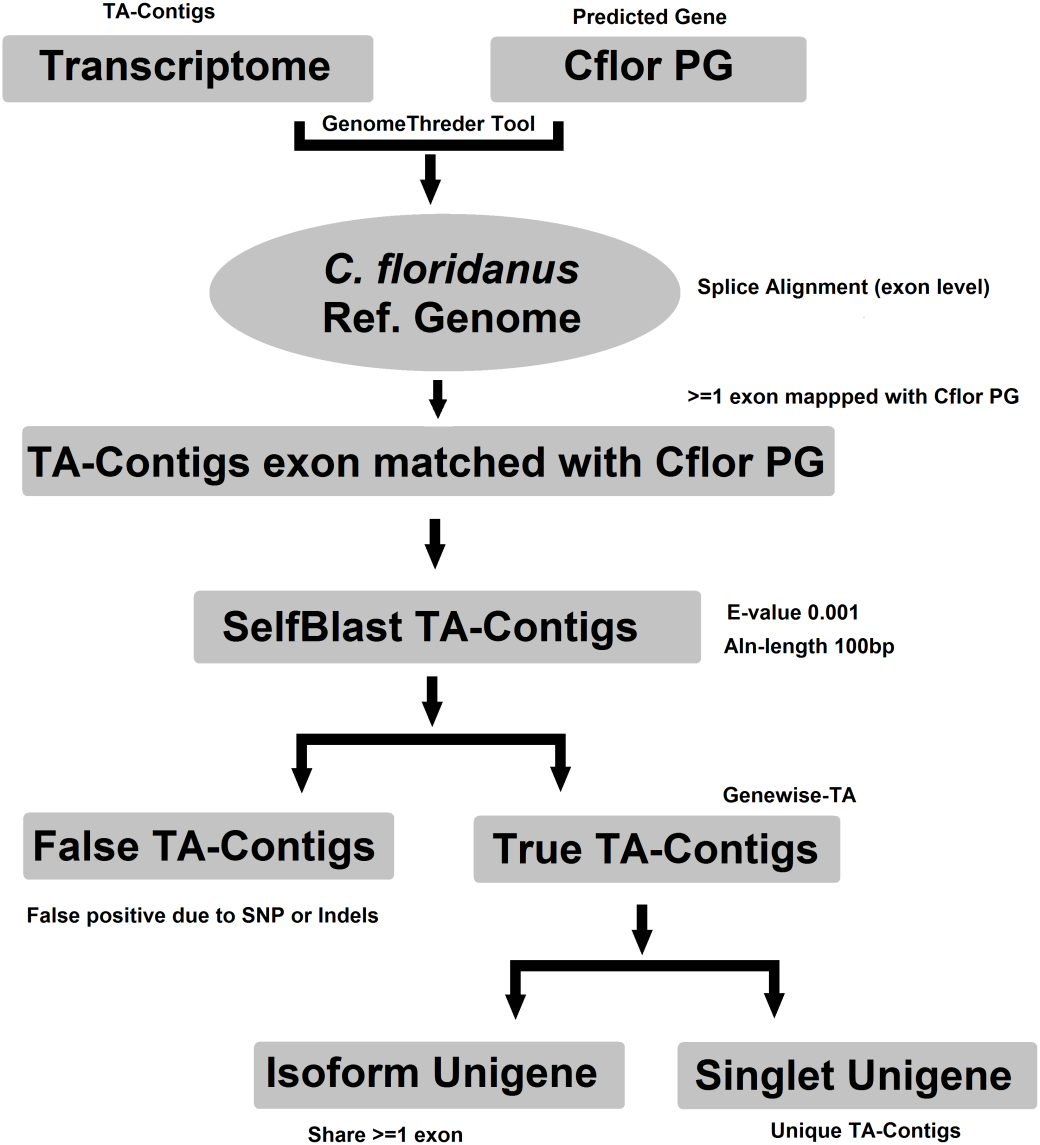
Workflow for compiling the Unigene-TA with isoform counts by contigs assignment to genes using alignment to proteins of related species.

Evidence-TA contigs were aligned to the genome of *C. floridanus* using GenomeThreader (Gremme et al., 2005) to obtain information on gene structures (number and location of exons) of assembled transcripts, and to estimate the total number of genes and isoforms included in the Evidence-TA. *C. floridanus* diverged from *F. exsecta* approximately 80 million years ago (Moreau et al., 2006). In order to resolve the gene structure, all spliced alignments of transcripts were combined to generate a consensus alignment, which also helped to uncover alternative splicing. Isoform contigs were grouped to the *C. floridanus* genes by the exon(s), to which they show the greatest similarity. Using the genome of closely related species, instead of self-BLAST, also allows joining non-overlapping contigs to the reference genes (Perl Script present in File S2). We also manually validated 300 probable isoforms to assess the quality of isoform prediction obtained with this workflow (Fig. 2). After this, the longest isoform identified by GenomeThreader was chosen for inclusion in the final TA containing only unique and non-redundant contigs, supported by biological evidence. These can be considered as expressed sequence tags (ESTs) of *F. exsecta* (Unigene-TA). The longest isoform was chosen, as it is likely to contain all or most of the gene exons and is, with some caution such as recent gene duplications, comparable to a predicted codon set extracted from a genome, and can be used in a similar manner in downstream applications. Genes with very high DNA sequence divergence rates may, however, have been lost during this process. The Unigene-TA transcripts were finally compared to Protein databases (NCBI, swissprot) using the blast2go tool (Conesa et al., 2005) for annotations of gene ontology.

## Results and Discussion

### Assemblies

In total, we obtained 322 M high quality reads. Based on ENCODE consortium recommendations, up to 100 million reads may be needed to successfully profile gene expression, with correctly detected isoforms in human data (The ENCODE Consortium, 2011). Since NGS technologies produce sequence data as short reads, and have a higher error rate (0.1–1%), a higher depth of sequencing is required for *denovo* assembly. Nevertheless, the optimal sequencing depth again depends on the aims of the experiment, non-uniformity of coverage in RNASeq, and sequencing error rate. For non-model organisms without a reference genome, the number of reads required should be scaled based on genome size, number of genes, and the total number of protein-coding genes. As the estimated number of the protein coding genes is somewhat smaller in ants than in humans (17,220 genes Oxley et al., 2014) compared to 19,836 in humans (GENCODE release 27, Harrow et al., 2012, and the total genome size much smaller (Tsutsui et al., 2008; Harrow et al., 2012), and our RNA-Seq data was obtained from different life stages and castes, we can therefore expect a reasonably high coverage, missing only transcripts with very low expression levels.

**Figure 3:**
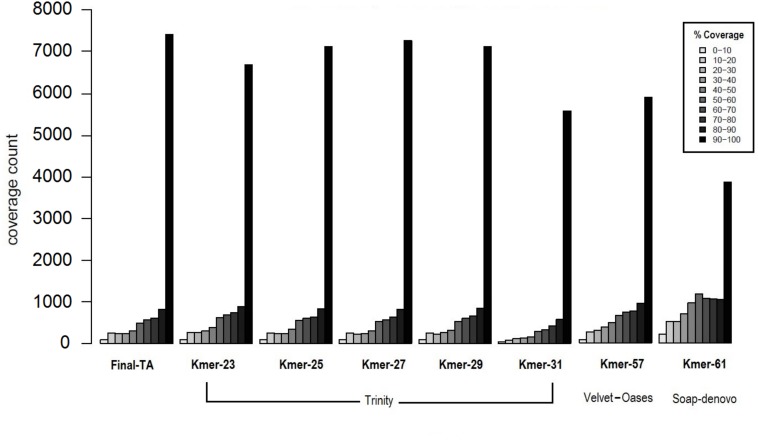
Coverage of contigs to *C. floridanus* predicted proteins. All k-mer assemblies produced by Trinity, and used to merge the Final-TA are shown, whereas only the assembly k-mers producing the largest fraction of >90% coverage are shown for Velvet-Oasis and SOAP-denovo. With respect to the transcripts that covered >90% of the *C. floridanus* proteins, nearly all Trinity k-mer assemblies outperformed Velvet-Oasis and SOAP-denovo, and the Final-TA yielded more transcripts than any individual k-mer assembly.

The transcriptome assemblers reported total assembly lengths of initial assemblies of up to 85–245 million bases (Mb), depending on software, with the maximum number of contigs generally between ca. 200 k and 300 k (File S1, Tables S1B–S1D). These are typical numbers for a *de novo* transcriptome assembly (O’Neil & Emrich, 2013; Nakasugi et al., 2014; Rana et al., 2016). However, these are inevitably, at some level, inflated by structural variation, such as SNP and indels, given the use of pooled RNA from individuals of many populations and colonies, and non-source species contaminant sequences (Johansson et al., 2013). A reduced contig number in the initial assembly could potentially be achieved by using individuals with less genetic variability. However, this is not possible in *F. exsecta,* where most colonies produce reproductive individuals of one sex only (Sundström, Chapuisat & Keller, 1996), and sampling the old queens in the spring precludes sampling their offspring later in the season. The general expectation that short k-mers produce more, but shorter contigs than long k-mers, held for all tested software. SOAPdenovo-trans and Velvet-Oases gave the highest N50, and average contig lengths using relatively long k-mers between 50–61 (2,167/681.7 and 2,133/835.8 bases, respectively; File S1, Tables S1B and S1C). Conversely, Trinity gave the highest N50 and average contig length with a shorter k-mer size *K* = 31 (2,863/2,032 bases, respectively; File S1, Table S1D). Another notable difference between the Initial-TA assemblies of the three assemblers resulted from reported non-ATGC characters (N’s), which formed up to 9.45% in the SOAPdenovoTrans assemblies and 0.017% of the Velvet-Oases assemblies, whereas the Trinity assemblies do not report any unknown bases (File S1, Tables S1B–S1D). We chose the best three assemblies (k-mer 57 of Velvet-Oases, k-mer 61 of SOAPdenovo-trans, k-mer 25 of Trinity) from each software, based on the number of reads used for assembly, N50, average contig length, and the number of ‘N’ bases. However, unlike in genome assemblies, where the aim is to maximize contig and scaffold lengths, TA’s are expected to contain smaller sequence fragments. Hence, to find the best method to produce TAs, we compared the coverage of TAs produced from each assembler to the closest annotated genome, as proposed by Haas et al. (2013). Subsequently 34% and 37% of the TA contigs produced by SOAPdenovoTrans and Velvet-Oases, respectively, but 67**%** of the TA contigs generated by Trinity, aligned to the predicted Protein set of *C. floridanus* with high confidence (100 bp alignment length and 70% sequence identity). There are 3,873 and 5,916 proteins of *C. floridanus,* that are represented by transcripts (TA contigs) with ≥90% alignment coverage, produced by SOAPdenovoTrans and Velvet-Oases, respectively (Fig. 3). Overall, the Trinity-assembled initial-TAs with different k-mers (range: 21–31) covered a higher number (range: 6,704–7,288) of *C. floridanus* proteins than different k-mer-specific Initial-TAs of the other assemblers (SOAPdenovoTrans and Velvet-Oases) with the same criteria. Trinity with k-mer 31, was an exception (5,592 *C. floridanus* protein only); this k-mer performed weakly in general, and only generated 14,661 transcripts (File S1, Tables S1D), i.e., only a 10th of the number generated by the other Trinity k-mers (range: 155,962–189,896 transcripts).

The lower alignment rates of contigs produced by SOAPdenovoTrans and Velvet-Oases, suggest that these methods produce more false positives, and incomplete assemblies than Trinity. In particular, contigs produced by SOAP include strings of N’s used for gap closing of read pairs. The number of genes identified by all assemblers (range: 10,916 to 11,123, with a total of 10,144 identified by all three assemblers), and those identified by one or two of them are shown in a Venn diagram (File S1, Fig. S1D). An approach for de novo transcriptome assembly that takes advantage of the assemblies of all different assemblers with various k-mer lengths is highly desirable, but it would also make datasets more complex (high heterozygosity, high error rate, high number of indels, contig orientation), and scaffolding would become computationally intractable. We therefore chose to only use the Trinity assembler as it is able to reconstruct the best full-length transcripts, with the ability to report also splicing variants. Our results agree with other studies that regard Trinity as the best transcriptome assembler over SOAPdenovoTrans and Velvet-Oases (Zhao et al., 2011; Nakasugi et al., 2014; He et al., 2015; Rana et al., 2016).

Biological reasons for the misalignment of contigs to *C. floridanus* proteins, including sequences that are highly variable, taxon restricted (orphans), or of non-source species origin (e.g., microbial contaminations, Johansson et al., 2013), are expected to affect all assemblers equally and not produce biases in the comparison. Trinity performed the best for our data, and produced 7,145 full length transcripts with more than 90% coverage with CflorPP proteins (File S1, Table S1E), consistent with its performance in previous comparisons of TA methods (Li et al., 2014; Chang, Wang & Li, 2014; Kiuchi et al., 2014; Balakrishnan et al., 2014; Davies et al., 2014). Given that Velvet-Oases has been found to perform nearly as well as Trinity in other studies (Tulin et al., 2013), it is likely that different assemblers and settings are optimal for different types of libraries, platforms, and read lengths. Initial-TA contigs from Trinity were chosen to build a combined assembly (Meta-TA) from different k-mer runs (File S1, Table S1F) by scaffolding overlapping contigs with Vmatch software (Abouelhoda, Kurtz & Ohlebusch, 2002). This procedure results in a non-redundant set of contig sequences originating from the initial k-mer specific Trinity assemblies. The merged Meta-TA assembly produced a total of 234,970 contigs (File S1, Tables S1G, S1H), that covered nearly 7,435 *C. floridanus* genes. This covers more than 90% of their protein length, which is 2–25% more than the length obtained from any single k-mer run of Trinity, including the default k-mer setting (Fig. 3). Thus, combining k-mer runs enhanced the average length, and length distribution of the contigs (File S1 Tables S1G, S1H).

**Figure 4:**
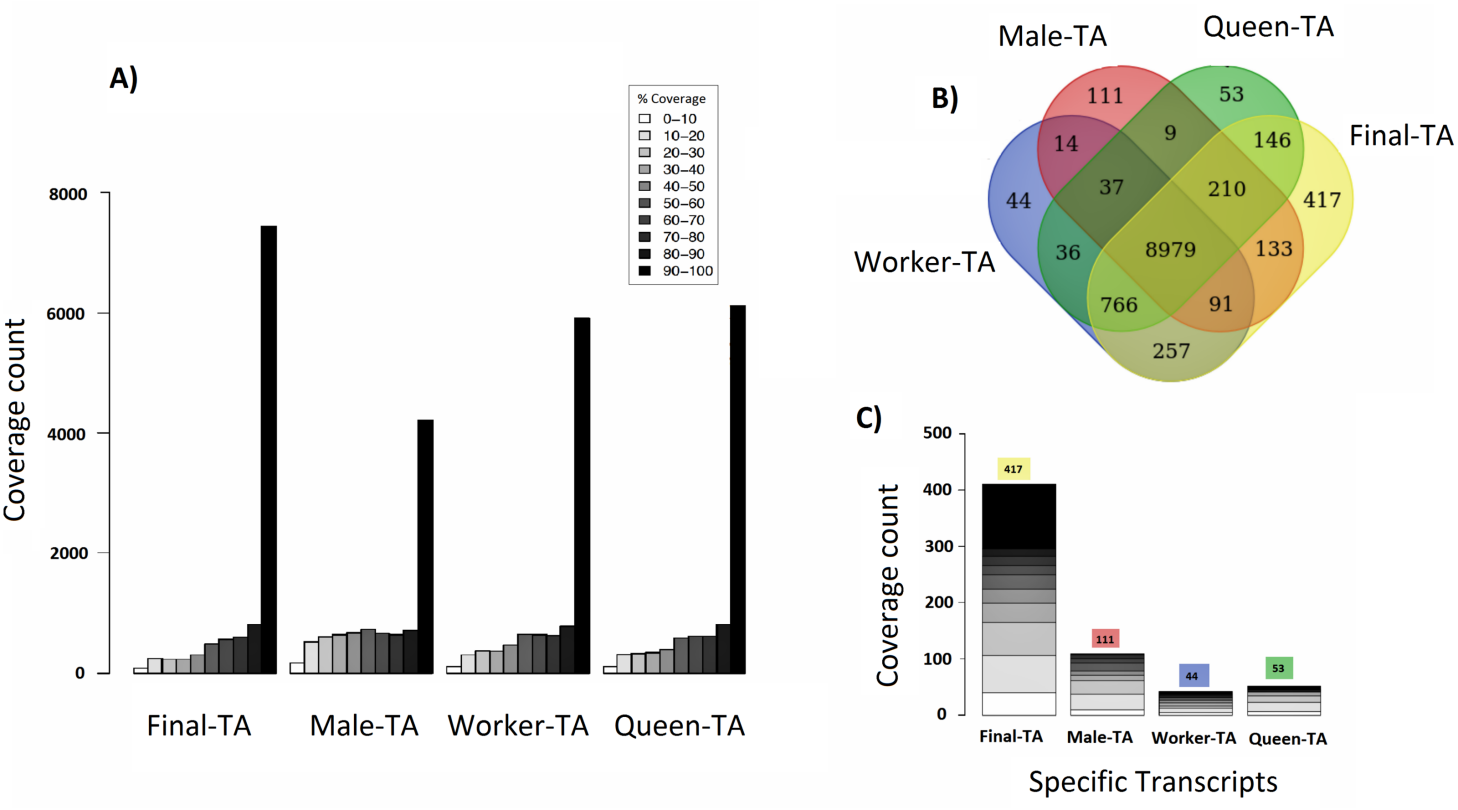
Coverage of contigs between castes. (A) Alignment of caste-specific assemblies to *C. floridanus* predicted proteins compared to the Final-TA, including all castes. (B) Overlap of transcripts aligning to *C. floridanus* proteins, by castes and the Final-TA. (C) Alignment coverage of contigs to *C. floridanus* assembled by caste or in the Final-TA.

We achieved the highest-quality transcriptome (Final-TA) by combining the output from the Trinity *de novo* assembler with different k-mers (range: 21–31). We also show that simultaneous assessment of a variety of metrics, not just focusing on the contig length or N50 value, is necessary to gauge the quality of the assemblies (File S1, Tables S1F and S1H, Fig. S1I). For transcriptome assembly, functional evaluation is more important than other parameters, like transcripts length or N50 values (Vera et al., 2008; O’Neil & Emrich, 2013). In addition, for polyphenic species like ants, it is important to sample individuals from all morphs, and from different developmental stages in order to obtain an adequate overview of expressed genes. For example, an assembly constructed with combined data (pooling males, queens and workers) covered nearly 7,435 *C. floridanus* genes (with 90% of their protein length), whereas an assembly of males, queens and workers, separately, each covered fewer genes with 90% coverage cut-off (4,215, 6,134 and 5,913 genes, respectively) (Fig. 4A). Also, a BUSCO transcriptome quality analysis using the Hymenoptera reference lineage showed that the Final-TA produced a higher number (3,936 genes) of full length transcripts than the specific caste assemblies (File S1 Fig. S1J).

The combined assembly (Final-TA) represents the maximum number of full length transcripts, which covers 10,999 *C. floridanus* genes from a total of 15,977 non-redundant proteins (CflorPP90), (File S1, Table S1H). Although the Final-TA covers only 69% of the *C. floridanus* genes, most transcripts share gene orthologies with other ant species, such as *A. echinatior*, *S. invicta*, *A. cephalotes*, *H. saltator*, *L. humile* and *P. parbatus* (Wurm et al., 2009). In total, the 42, 476 contigs in the Final-TA were annotated with 17,496 unique genes across all the 31 ant species available in the NCBI database, and 8,906 proteins top matches came from *C. floridanus* (File S1, Table S1J). In our final annotation the number of unique genes is within the range that we see in other sequenced ant genomes (range: 16,123–18,564; median: 17,220; Oxley et al., 2014). Out of 42,476 contigs, 29,041 share gene orthologies with seven ant genomes (*C. floridanus, A. echinatior*, *S. invicta*, *A. cephalotes*, *H. saltator*, *L. humile* and *P. parbatus*) sequences, and represent 8,375 conserved unique genes across them. The remaining 13,435 contigs showed orthologs unique to one ant species only and were absent in the other ant species.

### Caste-specific expressed transcripts

Separate assemblies of the castes showed that 8,979 *C. floridanus* genes (3,725 of which had 90–100% coverage, Fig. 4A), are shared across the castes (Fig. 4B), whereas 536 genes were unique to a caste or sex (133 in males, 257 in workers, 146 in queens), and 766 specific to the two female castes combined (Fig. 4C, Files S3A– S3D). A comparison of assemblies from different castes (male, queen, worker) reveals that the combined assembly has 417 caste-specific gene transcripts (Fig. 4C). We would have missed these gene transcripts if separate assemblies for each sample had been made, given differences in expression levels, and sequencing coverage for each specific caste. Using the Final-TA, we determined caste and sex -specific genes, and found that 766 genes were only expressed in queens and workers (File S3D), whereas 133 of the genes were exclusively expressed in males. Among the female-specific genes, several are involved in biosynthetic processes, such as macromolecule, and lipid biosynthesis, or biopolymer catabolic process (File S1, Fig. S1K). In addition, many are associated with cell communication and signal transduction. The queen-specific genes comprise categories, such as translation, nucleus, carbohydrate binding, nucleic acid binding, system process, ATP-, DNA- and RNA binding. Of the 133 male-specific genes, 42 matched those reported previously in the honey bee sperm proteome (Zareie et al., 2013). This subset contains a significant number of proteins that are predicted to act in enzyme regulation or in nucleic acid binding and processing (Zareie et al., 2013).

### Transcriptome functional annotation & analysis

From the Meta-TA, we selected contigs at least 200 bp in length for annotation, and gene ontology assessment in the Evidence-TA. This resulted in 120,212 contigs in total, including predicted isoforms (Genbank accession number: GFLV00000000). Of these, 39,262 (32.7%) contigs were annotated with available ant genomes including the two closest relatives *C. floridanus* and *L. niger*, and 5010 (4.2%) to other insects (File S4, NCBI update 2015). These comprise the Evidence-TA (Fig. 1). The top hit species distribution from a BLAST analysis, including the NR database update 2017, shows that most annotations come from *C. floridanus* (20,295 transcripts), *L. niger* (7,314 transcripts), *C. biroi* (1,305 transcripts), *L. humile* (1,227 transcripts) and *S. invicta* (1,136 transcripts) (File S1, Fig. S1H).

Of the sequences excluded from the Evidence-TA, 1,847 (1.5% of the contigs in Meta-TA) were aligned to micro-organisms, such as bacteria, viruses, and fungi, or other non-species genes (File S3 and Johansson et al., 2013). However, the majority (61.6%) of the contigs (74093) were not aligned to any available databases including ants, other eukaryotes, or their pathogens. In comparison, our annotation rate is within the range reported (20–40%) for several other *de novo* transcriptome assemblies in non-model species (Vera et al., 2008; Wang et al., 2010; Fraser et al., 2011; Hou et al., 2011; Du et al., 2012), and considerably higher than the rate of unique genes annotated in an earlier study of *F. exsecta* (Badouin et al., 2013). Most of the unannotated contigs are shorter (mean 486.7 bp; median 343 bp) than those annotated (mean 1,965 bp and median 1,510 bp, respectively). Only 29% of the unannotated contigs were longer than 500bp. The total size of the annotated contigs is 87 MB base pairs, whereas the unannotated ones only comprise 37 MB base pairs, despite being more numerous. This suggests that many of the unannotated sequences are fragmented.

Our chosen homology-based annotation strategy leads to an inherent bias so that only conserved genes, with identifiable orthologs, are included in further analyses. A subset of these contigs may represent sequences that have gone through high diversification since the divergence between *Formica* and *Camponotus* (Moreau et al., 2006), or other ant genes, not preserved in the phylogenetic lineage leading to *Camponotus.* Given the average rate of orphan genes found in arthropod genomes (Wissler et al., 2013), and the estimation that every eukaryotic genome contains 10–20% of taxonomically-restricted genes (TRGs) without any significant sequence similarity to genes of other species (Johnson & Tsutsui, 2011), our rough estimate is that approximately 1,000 true genes were lost when we filtered out the unannotated contigs. These should comprise a minority of the unannotated transcripts, whereas the majority should be expressed pseudogenes (Kalyana-Sundaram et al., 2012), previously uncharacterized transposons or other mobile genetic elements (Dion-Cote et al., 2014), micro-organisms such as viruses Johansson et al., 2013, regulatory RNA species of protein-coding genes such as micro-RNA (miRNA) precursors and regulatory long non-coding RNA (lncRNA) (Morris & Mattick, 2014), and/or prediction artefacts. The predominantly short lengths of the unannotated contigs support this interpretation. A genome sequence of *F. exsecta,* or close relatives, would be necessary for investigating whether the unannotated sequences are indeed protein-coding genes, prediction artifacts, or non-coding RNA. A caste specific role of TRGs has been suggested (Sumner, Pereboom & Jordan, 2006; Ferreira et al., 2013; Feldmeyer, Elsner & Foitzik, 2014; Harpur et al., 2014), but is beyond the scope of this study.

**Figure 5:**
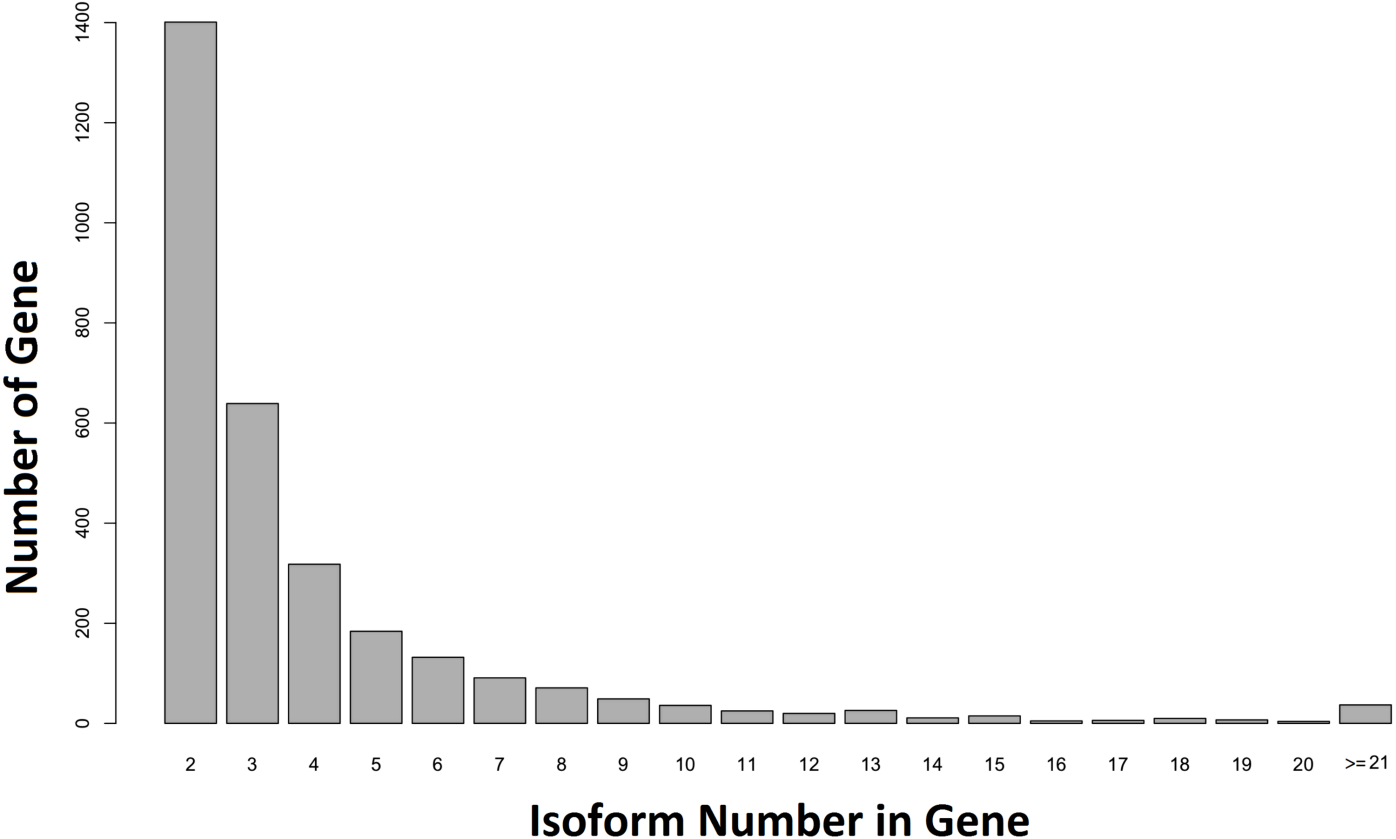
Number of isoforms detected in non-singlet genes. More than one isoform was found in 44.71% (*n* = 3, 087) of the annotated genes against *C. floridanus*.

When our contigs were aligned to the *C. floridanus* genome, and the Unigene-TA constructed, our data contained 6,894 annotated unique genes, with 3,807 genes in singlets (File S5), and 3,087 genes distributed across several isoforms (range 2–84, average = 4.1, s.d. = 4.2, File S6; Fig. 5). In comparison, in *Drosophila melanogaster* approximately 60% of the multi-exon genes were estimated to contain several isoforms (Stolc et al., 2004). As the majority of genes (65%) with many isoforms encodes two or more protein products, alternative splicing generates considerable protein diversity in *Drosophila* (Misra et al., 2002). Our data suggests that the ca. 8,000 unique hits to other insect genomes found an earlier transcriptome of the *F. exsecta* (Badouin et al., 2013) is an overestimate due to isoforms treated as separate genes. This demonstrates that the steps from Evidence-TA contigs (containing isoforms separately) to Genewise-TA (isoforms combined to genes) are important for the correct estimation of gene numbers.

Considering isoforms as separate genes could potentially bias downstream analyses such as GO-enrichment. A total of 23,016 sequences from the Final-TA were assigned to one or more gene ontology using Blast2Go. Of those sequences, 11,025 belonged to Unigene-TA along with isoforms and 4,560 to Unigene-TA (File S1, Fig. S1I). The percentages of gene counts for each gene ontology term were significantly different between the Unigene-TA sets with and without isoforms (*t* = 3.4, *df* = 11, *p* = 0.006) in the cellular component GO category. However, no differences were found in the biological process (*t* =  − 0.95, *df* = 16, *p* = 0.36), and molecular function (*t* = 0.088, *df* = 9, *p* = 0.93) GO categories. As expected, the frequencies of GO terms obtained from the Final-TA (i.e., the whole data set containing all isoforms) closely follow those found in an earlier paper on the same species (Badouin et al., 2013). In both studies the most frequent 12 GO terms are the same within the category biological processes. In addition, the two most highly represented GO terms for molecular function coincide with those detected by Badouin et al. (2013).

### Gene families and analysis

The evolution of many gene families is thought to be affected by the evolution of eusociality (Simola et al., 2013). In particular, social insects make intensive use of chemical cues in communication and recognition (Bradbury & Vehrencamp, 2011; Richard & Hunt, 2013; Wyatt, 2013), and have therefore been predicted to have diversified in this respect, compared to solitary insects (Bradbury & Vehrencamp, 2011; Wyatt, 2013). We detected 12 chemosensory protein (CSP) genes, and three odorant binding protein (OBP) genes, in which the number of isoforms varies between 1 and 10 (average = 2.2, s.d. = 2.0, File S7). This falls within the range found in other species of ants, in which the number of functional CSP genes ranges from 11 to 21 depending on species (Vieira & Rozas, 2011; Zhou et al., 2013; Kulmuni & Havukainen, 2013). This is, however surprisingly low, given that solitary insects, such as the flour beetle *Tribolium castaneum* (19 genes), the silkworm *Bombyx mori* (22 genes), and the locust *Locusta migratoria manilensis* (more than 70 genes), show gene numbers in the upper range or much higher than those found in social insects. Thus, the evolution of gene families, and the genetic underpinning of odour cue diversity in social insects, is likely more complex, and should be considered together with isoform variation across taxa. Indeed, presence of multiple isoforms has been demonstrated for several CSP genes. For example, seven CSP isoforms were found in individual *Schistocerca gregaria* legs (Angeli et al., 1999), 14 CSP isoforms in *Locusta migratoria* (Ban et al., 2003), seven OBP isoforms in the hemolymph of *Tenebrio molitor* (Graham et al., 2003) larvae, and 38 isoforms in the fly *Drosophila melanogaster* (Graham & Davies, 2002).

The number of immune genes in ants is predicted to depend on the extent of social immunity and hygiene behaviours (Cremer, Armitage & Schmid-Hempel, 2007), which may reduce the need for a high number of immune genes (Evans et al., 2006). Indeed, in the honey bee, many immune-gene families appear to be depauperate, when compared to solitary insects (Evans et al., 2006; Werren et al., 2010). Our transcriptome contained only some of the antimicrobial-peptide coding genes described previously in ant genomes (77 unique genes, up to 10 isoforms, average = 1.4, s.d. = 1.4, File S8). This may partly be explained by the fact that none of the samples were purposely challenged with pathogens before preparation. However, given the variety of pathogens (pathogen pressure) present in natural colonies (Baird, Woolfolk & Watson, 2007; Boots et al., 2012; Duff et al., 2016), these genes might not be expressed at the specific time point samples were collected, but may be present in the genome. In general, any conclusions about absence of genes from the genome, and gene family sizes in general should be treated with caution when based on expression data only.

Vitellogenins (Vg) comprise a third gene family (Wahli et al., 1981; Sappington, 2002; Tufail & Takeda, 2008), which has diversified among many ants, and has many genes with caste biased expression patterns (Wurm, Wang & Keller, 2010; Corona et al., 2013; Morandin et al., 2014; Salmela & Sundström, 2017). Here we report five copies of Vg and one Vg receptor (File S9), and their isoforms Vitellogenin-like-B (four isoforms), Vitellogenin-like-D (two isoforms), Vitellogenin receptor (four), and only one isoform each of Vitellogenin-like-A, Vitellogenin-like-C, and the conventional Vitellogenin genes. None of these were specific to any sex or caste. Earlier work on *F. exsecta,* where the same reads of queens and workers were applied, found altogether four vitellogenin gene orthologues, (Vitellogenin, Vitellogenin-like-A, Vitellogenin-like-B, Vitellogenin-like-C) each with specific expression patterns and great structural variation (Morandin et al., 2014). Vitellogenin-like-D gene is shortest among all Vg’s and missed in earlier work due to unavailability of annotation in initial stage and its partial assembly. As vitellogenins are known to have both antioxidant, antimicrobial and storage functions ( Havukainen, Halskau & Amdam, 2011), in addition to their key role in caste regulation in e.g., the honeybee (Piulachs et al., 2003;  Havukainen, Halskau & Amdam, 2011; Salmela & Sundström, 2017), further work on isoform expression could shed light on the role of the different vitellogenins in the of caste phenotypes (Wurm, Wang & Keller, 2010; Corona et al., 2013; Morandin et al., 2014).

Genes related to epigenetic regulation are of current interest for their role in generating and maintaining caste differences in social insects. Genomic analyses have revealed the evolutionary persistence of DNA methylation across Hymenoptera (Glastad et al., 2011), in contrast to e.g., Diptera, where methylation is diminished (Marhold et al., 2004). Our transcriptome data reveals all DNA methyltransferases required for CpG methylation, including DNMT1 for maintaining methylation patterns, and DNMT3, which is responsible for *de novo* methylation. These DNMTs have also been found in other ant species (Bonasio et al., 2010; Suen et al., 2011; Smith et al., 2011b; Wurm et al., 2011; Nygaard et al., 2011; Oxley et al., 2014; Schrader et al., 2014). DNMT2 RNA methyltransferase was recently reported to be required for the establishment and hereditary maintenance of methylation patterns in mice (Kiani et al., 2013). Thus, the epigenetic inheritance and its regulation is undoubtedly more complex than understood today (see also e.g., Wang et al., 2013; Yan et al., 2014). Similarly to e.g., mice (Lin et al., 2000), we found splice variants in DNMT genes (up to 4 isoforms cf. File S7), and given the role of methylation in caste differentiation in social insects (Glastad et al., 2011), the role of splice variants is certainly an interesting direction for more studies.

## Conclusions

Given the rapid advances in transcriptomics of non-model organisms, with ever more comparative studies being carried out, it is all the more important that the transcriptomes are reliable. In this study we provide insights into essential criteria that should be taken into account for a reliable transcriptome assembly without a reference genome. First, we find that analyses relying on single life stages or morphs is likely to miss some genes due to low sequencing depth or expression at specific stage. Second, care should be taken in choosing the assembly methods, and different methods should be compared in order to find which one performs the best for a given species data set. In addition to this, filtering with a reference genome of a related species is necessary for quantifying transcript abundance, and doing functional and structural annotation of transcripts, so that isoforms are not analyzed as unique genes in downstream applications. This necessarily comes with the cost of losing orphan genes from the data. While the study of orphan genes is obviously valuable, for the purposes of facilitating species comparisons on various taxonomical scales, we opted for analyzing genes where annotations are available so that we can be sure we are working with true genes. Identifying isoform variation from unique genes allows studies on structural mRNA diversity in genes of interest, which may be evolutionarily more important than variation in expression levels.

##  Supplemental Information

10.7717/peerj.3998/supp-1 File S1 Transcriptome analysis data information with assembly statistics

10.7717/peerj.3998/supp-2 File S2 Perl script used for the isoform detections

10.7717/peerj.3998/supp-3 File S3 List of caste and sex specific genes from Final-TA assembly

10.7717/peerj.3998/supp-4 File S4 Species distribution of Meta-TA transcripts annotated with ants, other insects, bacteria, fungi and virus

10.7717/peerj.3998/supp-5 File S5 Singlet genes in the Unigene-TA assembly

10.7717/peerj.3998/supp-6 File S6 Genes with probable isoforms in the Unigene-TA assembly

10.7717/peerj.3998/supp-7 File S7 Chemical communication and DNA methylation genes and their probable isoform numbers from the Unigene-TA assembly

10.7717/peerj.3998/supp-8 File S8 Immune function genes and their isoform numbers from the Unigene-TA assembly

10.7717/peerj.3998/supp-9 File S9 List of Vitellogenin genes and their probable isoform number from the Final-TA assembly

## Additional Information and Declarations

### Data Availability

The following information was supplied regarding data availability: BioProject: PRJNA213662
BioSample: SAMN03799245
Figshare: https://doi.org/10.6084/m9.figshare.4877360.
