# Supplementary material for: Transcriptome sequencing reveals high isoform diversity in the ant Formica exsecta
Source: PeerJ. 2017 Nov 21;5:e3998. doi: 10.7717/peerj.3998 (PMC5701548; doi:10.7717/peerj.3998)

Sequencing data for assemblies

The raw paired-end sequence data in FASTQ format is deposited in the National Centre for Biotechnology Information's (NCBI) Short Read Archive (SRA) database under the Bioproject accession number PRJNA213662. Before proceeding with the *de novo* assembly, we applied quality control and filtering of sequencing reads. The FastQC indicated that the reads consist to more than 90 % of high quality (>Q20) bases in both the forward, and the reverse (paired-end) reads (Table S1-A). Low quality bases were mostly located at the end of reads, and we subsequently trimmed 14 bases from end of raw reads. No adapter contamination was present.

For running *de novo* assemblies with different assembly programs, we combined the seven sequencing read libraries, forward and reverse reads separately, irrespective of life stage or caste. The pooled libraries are denoted as BGI_Read1.fq (forward reads) and BGI_Read2.fq (reverse reads).

Table S1-A: Summary of raw RNASeq data.

Caste/Sex	Sequencing Library	Raw Data (n*)	(Paired=n x 2**)	Bases before trimming (90bp length)	Bases after trimming (76 bp length)
Queen	Lib 1- Old Queen	53982965	107965930	9716933700	8205410680
	Lib 2- New Queen	40511938	81023876	7292148840	6157814576
	Lib 3- Queen Cocoon	39009869	78019738	7021776420	5929500088
Worker	Lib 4- Old Worker	51343611	102687222	9241849980	7804228872
	Lib 5- New Worker	41084727	82169454	7395250860	6244878504
	Lib 6- Worker Cocoon	41226923	82453846	7420846140	6266492296
Male	Lib 7- Male (Mix)	54979146	109958292	9896246280	8356830192

* Forward reads only.
** Paired end reads (Read forward +Read reverse).

Transcriptome assemblers and assembly statistics

Filtered high quality reads were assembled into contigs with four assembly softwares (SoapdenovoTrans (127mer version 1.0: Nov 12th, 2011), Velvet-Oases (Version 0.2.06), Trinity (Release 2012-05-18)), each with a range of k-mer/Hash lengths. Transcriptome assembly statistics were calculated for each k-mer-based assembly.

a) SoapdenovoTrans

Table S1-B. The assembly summary statistics of transcripts constructed using k-mers 21 -71 in the SoapdenovoTrans assembler. K-mers highlighted in grey indicate the best k-mer assembly based on the N50 value, average contig length, Non-ATGC characters, and contig numbers.

**k-mer length:**	**k-mer 21**	**k-mer 29**	**k-mer 41**	**k-mer 51**	**k-mer 55**	**k-mer 57**	**k-mer 61**	**k-mer 71**
Contigs generated	221372	234748	174919	137965	117924	107707	84014	30828
Maximum Contig length	43380	43043	39641	32863	37175	26418	46263	21401
Minimum Contig length	100	100	100	100	100	100	100	100
Average Contig length	595.011	552.166	643.999	671.008	681.741	684.63	719.187	643.451
Total Contig length	131718775	129619871	112647720	92575663	80393572	73739396	60421741	19836301
Total number of Non-ATGC characters	10081991	8532164	9108683	8664316	7562327	6943206	5710006	2144924
Percentage of Non-ATGC characters	0.0765418	0.0658245	0.0808599	0.0935917	0.0940663	0.0941587	0.0945025	0.108131
Contigs 100 bp	2476	3485	406	3547	332	197	104	5
Contigs > 100 bp	218896	231263	174513	134418	117592	107510	83910	30823
Contigs > 500 bp	55587	52373	44810	36699	32442	30159	25582	12115
Contigs > 1 Kb	27859	26949	24848	21902	19766	18569	16020	5694
Contigs > 10 Kb	470	482	443	325	193	152	90	6
Contigs > 100 kb	0	0	0	0	0	0	0	0
N50 value	1549	1559	1920	2151	2167	2121	2087	1050
N80 value	360	318	378	455	484	491	535	381
Soapdenovo log N50	1549	1559	1920	2151	2167	2121	2087	1050
Number of reads assembled	632168668	633652680	614038569	588399963	574779873	570343228	556522018	457068498
Total number of reads used for assembly	644278358	644278358	644278358	644278358	644278358	644278358	644278358	644278358
Percentage of reads assembled	98.1204258	98.35076285	95.30640931	91.3269794	89.21297245	88.5243499	86.379127	70.942705

Figure S1-A. Comparative summary statistics of transcripts constructed using SoapdenovoTrans assembler.

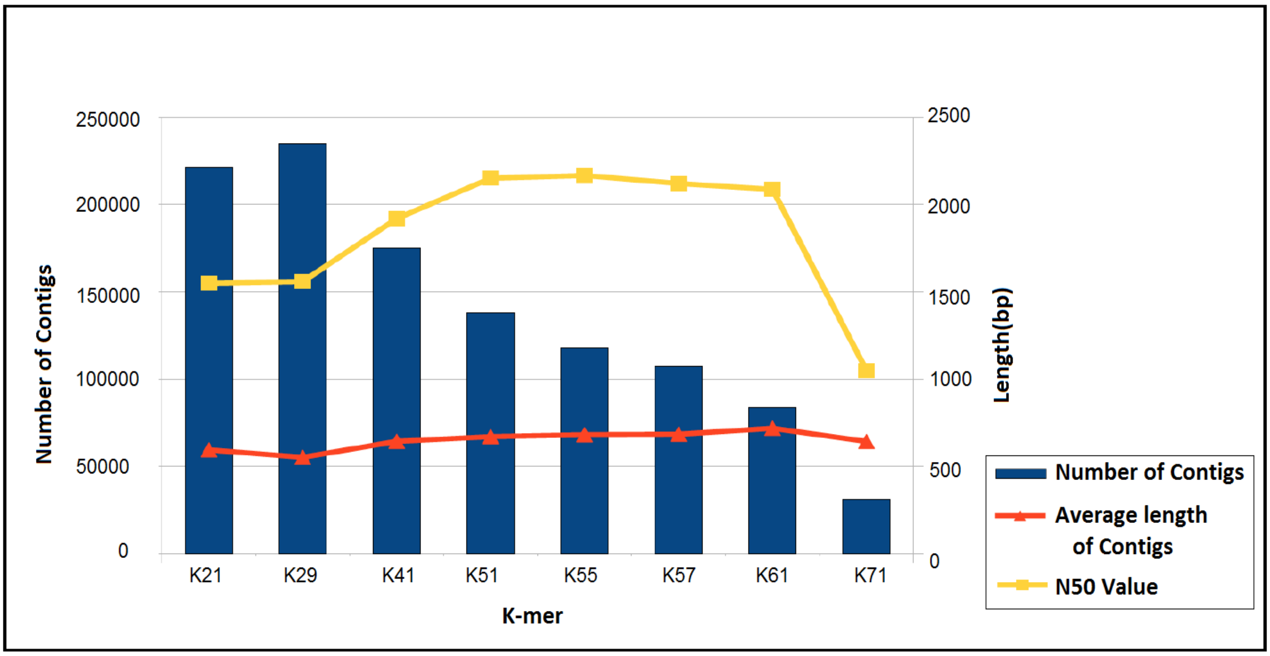

b) Velvet-Oases

Table S1-C. The assembly summary statistics of transcripts constructed using k-mers 51 -75 in the Velvet-Oases assembler. K-mers highlighted in grey indicate the best k-mer assembly based on the N50 value, average contig length, Non-ATGC characters, and contig numbers.

**k-mer length**	**k-mer 51**	**k-mer 53**	**k-mer 55**	**k-mer 57**	**k-mer 61**	**k-mer 65**	**k-mer 71**	**k-mer 75**
Contigs generated	157016	138446	292608	107696	83035	63668	40825	1458
Maximum Contig length	14843	18303	45775	23398	29174	22245	10911	5614
Minimum Contig length	100	100	96	100	100	100	100	125
Average Contig length	856.043	902.629	835.824	931.935	882.861	772.157	459.285	366.839
Total Contig length	134412440	124965370	244568859	100365649	73308389	49161675	18750300	534851
Total number of Non-ATGC	33659	27316	361066	17376	13543	7318	840	85
Percentage of Non-ATGC	0.000250416	0.000218589	0.00147634	0.000173127	0.00018474	0.000148856	4.47993e-05	0.000158923
Contigs 100 bp	158	117	2347	34	12	2	2	0
Contigs > 100 bp	156858	138329	290260	107662	83023	63666	40823	1458
Contigs > 500 bp	69177	60612	107826	46623	36103	26671	9914	184
Contigs > 1 Kb	41094	37856	66551	30167	22262	14736	2894	36
Contigs > 10 Kb	98	80	445	77	42	19	2	0
Contigs > 100 kb	0	0	0	0	0	0	0	0
N50 value	1681	1882	2133	1988	1783	1372	502	343
N80 value	574	624	610	647	573	457	281	252
Number of reads assembled	409355474	415263087	417977881	416373370	412989888	402698569	365045735	179887306
Total no. of reads used for assembly	644278358	644278358	644278358	644278358	644278358	644278358	644278358	644278358
Percentage of reads assembled	63.5371	64.45	64.8753	64.6263	64.1012	62.5038	56.6596	27.9207

Figure S1-B. Comparative summary statistics of transcripts constructed using Velvet-Oases assembler.

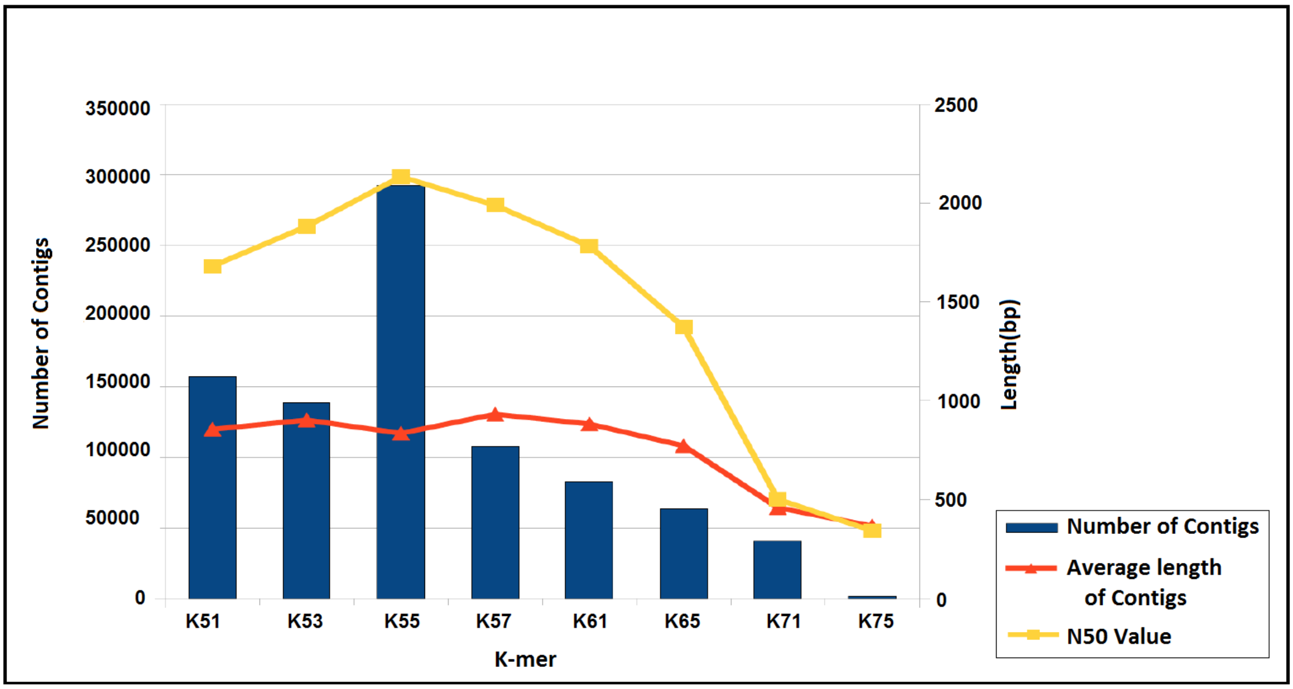

c) Trinity

Table S1-D. Assembly summary statistics of transcripts constructed using k-mers 23-31 in Trinity. K-mers highlighted in grey indicate the best k-mer assembly based on the N50 value, average contig length, Non-ATGC characters, and contig numbers.

k-mer length	k-mer 23	k-mer 25	k-mer 27	k-mer 29	k-mer 31
Contigs generated	155962	171926	186711	189896	14661
Maximum Contig length	17869	28268	23688	28226	23628
Minimum Contig length	101	101	101	101	101
Average Contig length	390.735	786.198	996.127	1051.48	2032.45
Total Contig length	60939816	135167843	185987849	199672087	29797811
Total number of Non-ATGC	0	0	0	0	0
Percentage of Non-ATGC	0	0	0	0	0
Contigs 100 bp	0	0	0	0	0
Contigs > 100 bp	155962	171926	186711	189896	14661
Contigs > 500 bp	25412	53175	70444	75466	13034
Contigs > 1 Kb:	11881	35626	50693	54831	9995
Contigs > 10 Kb	32	287	455	521	97
Contigs > 100 kb	0	0	0	0	0
N50 value	705	2417	2818	2919	2863
N80 value	217	619	985	1069	1506

Figure S1-C. Comparative summary statistics of transcripts constructed using Trinity assembler.

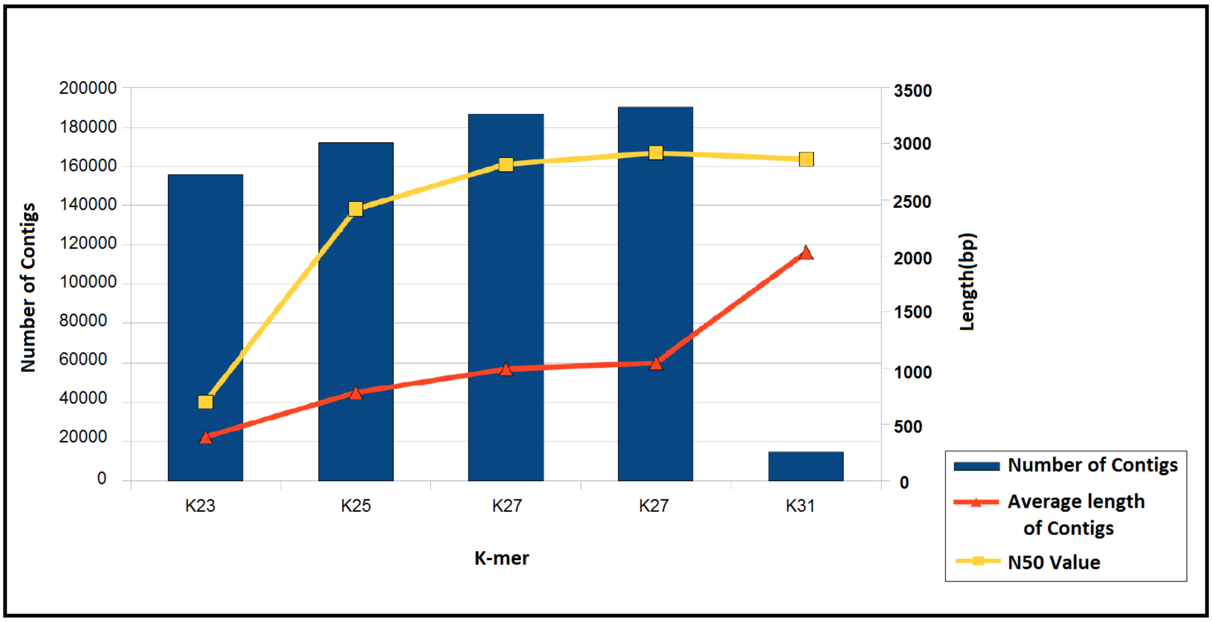

Coverage of best assemblies on *Camponotus floridanus* genes

To select the best assembler with an orthology-based approach, we aligned the technically (based on N50, average length, contigs) best assembly from each assembler to *Camponotus floridanus* proteins (CfloPP90), and calculated the coverage distribution for each. Trinity yielded the greatest number of transcripts aligned with high coverage (>90%; N=7145 vs 5916 and 3873), and was selected as the basis for the Meta-TA assembly.

Table S1-E. Coverage distribution of the best k-mer transcript assembly for each aligner to *C. floridanus*.

Cflor90 Coverage	Trinity k-mer 25	Velvet-Oases k-mer 57	Soapdenovo k-mer 61
0-10	86	74	205
10--20	254	267	513
20-30	230	306	514
30-40	238	379	702
40-50	346	487	962
50-60	551	656	1181
60-70	601	745	1070
70-80	630	769	1063
80-90	835	950	1040
90-100	7145	5916	3873
**Total no. genes covered**	**10916**	**10549**	**11123**

Figure S1-D. The *C. floridanus* protein-coding genes covered by the best k-mer assemblies of Trinity, Velvet-Oases and Soapdenovo-trans.

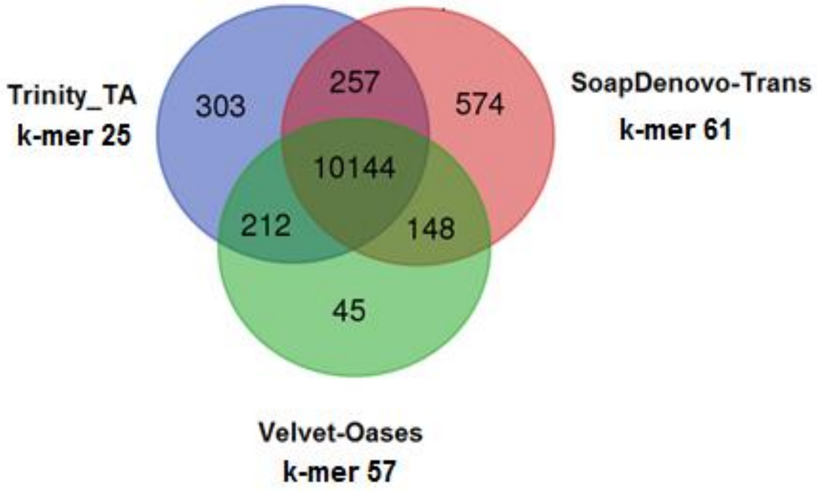

Table S1-F. Performance of Trinity assemblies based on different k-mers, measured by coverage distribution of the *Compontus floridanus* proteins (CflorPP90) using BLAST.

Cflor90 Coverage	Trinity k-mer 23	Trinity k-mer 25	Trinity k-mer 27	Trinity k-mer 29	Trinity k-mer 31
0-10	91	86	84	88	36
10--20	261	254	249	244	75
20-30	264	230	228	222	111
30-40	300	238	234	261	124
40-50	381	346	307	317	161
50-60	617	551	524	533	290
60-70	681	601	563	601	332
70-80	738	630	635	665	418
80-90	880	835	823	848	575
90-100	6704	7145	7288	7144	5592
Total no. genes Covered	10917	10916	10935	10923	7714

Figure S1-E. Coverage distribution of *Camponotus floridanus* proteins (Cflor90PP) against Trinity transcripts from different k-mer specific assemblies.

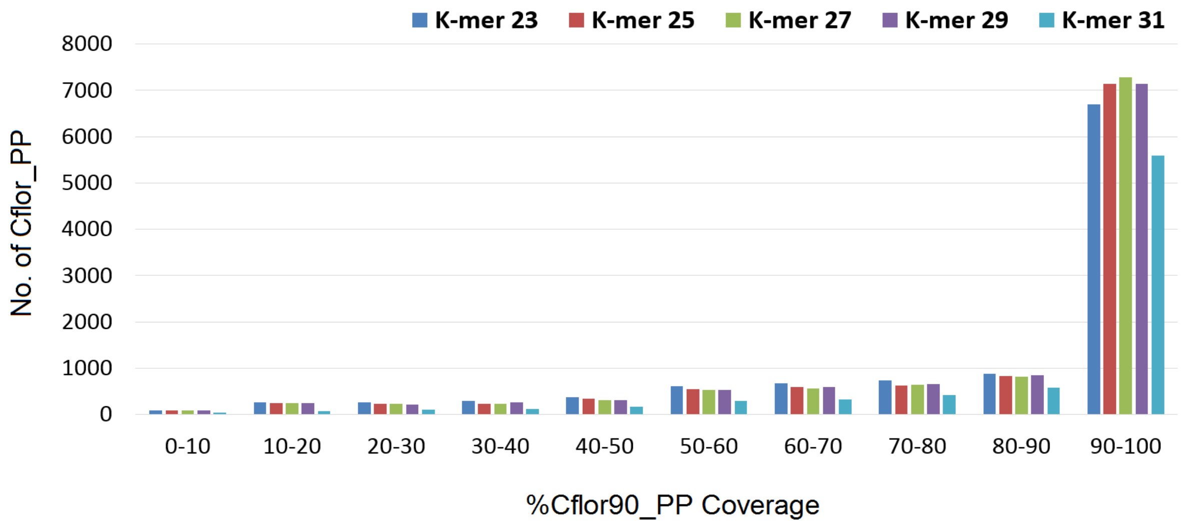

Figure S1-F. Overlap between full-length Cflor90PP proteins from different k-mer specific Trinity assemblies.

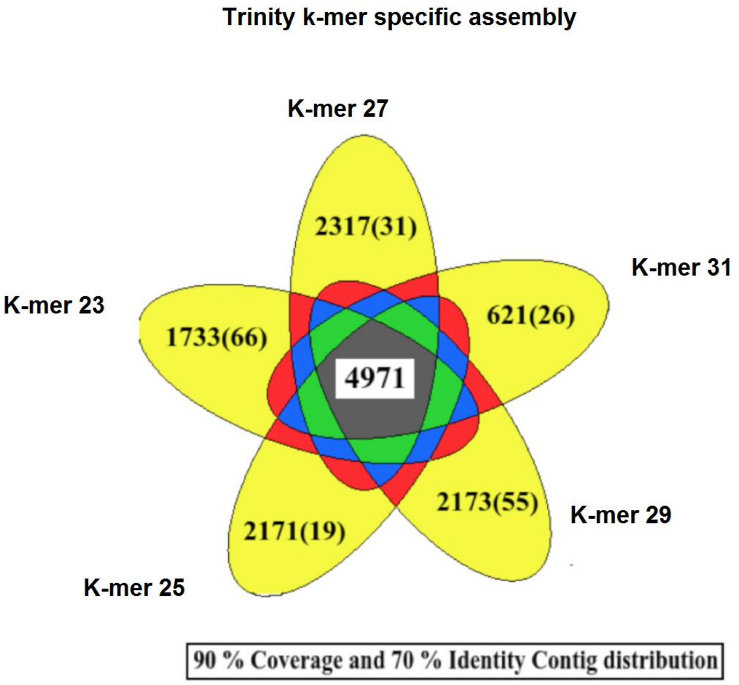

Meta-TA assembly

To achieve a high coverage transcriptome, we combined the output from all Trinity k-mer assemblies (k-mer 23, 25, 27, 29, and 31) using Vmatch. In addition to combining genes assembled by different k-mer-specific assemblies, collapsing the k-mer specific assemblies removes redundancy in the transcript data.

Table S1-G. Summary statistics of the Meta-TA assembly.

Meta-TA assembly QC	
Contigs generated	234970
Maximum Contig length	28268
Minimum Contig length	101
Average Contig length	930.907
Total Contig length	218735222
Total number of Non-ATGC characters	0
Percentage of Non-ATGC characters	0
Contigs 100 bp	234970
Contigs > 100 bp	150411
Contigs > 500 bp	87912
Contigs > 1 Kb	60056
Contigs > 10 Kb	534
Contigs > 100 kb	0
N50 value	2583
N80 value	819

Table S1-H. Coverage distribution of the Meta-TA assembly with the Cflor90 protein dataset. The Meta-TA assembly able to cover in total 10999 *Camponotus floridanus* proteins without any coverage cut-off.

Cflor90 Coverage	Meta-TA assembly (Vmatch)
0-10	86
10-20	246
20-30	230
30-40	230
40-50	299
50-60	488
60-70	567
70-80	606
80-90	812
90-100	7435
Total	10999

Annotation of the Meta-TA assembly

To annotate the assembled Meta-TA contigs we performed a BLAST search on the NCBI non-redundant protein (nr), and non-redundant nucleotide (nt) databases. Only Meta-TA contigs (transcripts) annotated to ants and other insects, which indicates that they are true protein coding genes, were accepted to the Final-Assembly.

Table S1-I. List of major taxa to which most transcripts sequeces were aligned during BLAST step (>=100bp Transcripts)

**Taxa**	**TA Contigs**
Ants	42476
Insects	5524
Bacteria	1528
Fungi	309
Virus	70
Nematode	844
Arthropods	42
Plants	116
Other Eukaryotes	534
Total	51443

Figure S1-G. Venn diagram of taxon distributions to which most transcripts sequeces were aligned during BLAST step (>=100bp Transcripts).

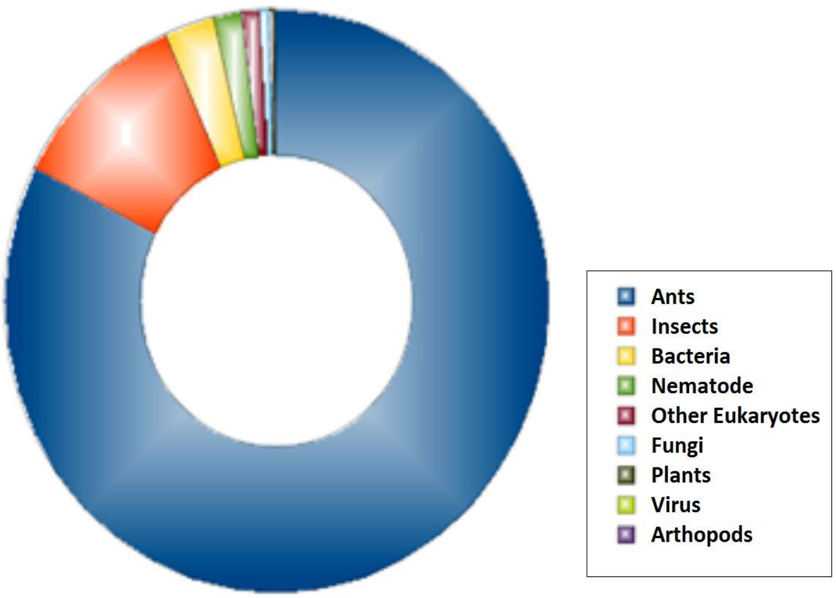

Table S1-J. The ant species to which most transcript sequences were aligned during the BLAST step(NCBI Database update 2015).

Species	TA Contigs	Unique Proteins
Camponotus floridanus	27111	8906
Acromyrmex echinatior	2363	1397
Solenopsis invicta	2891	1477
Atta cephalotes	2944	1768
Harpegnathos saltator	1913	1052
Linepithema humile	2561	1446
Pogonomyrmex barbatus	2620	1409
Other ants	73	41
Total	42476	17496

Figure S1-H: Top Hit- species list in the Final-TA (>=200bp Transcripts) with the NCBI non-redundant protein database (NCBI Database Update 2017).

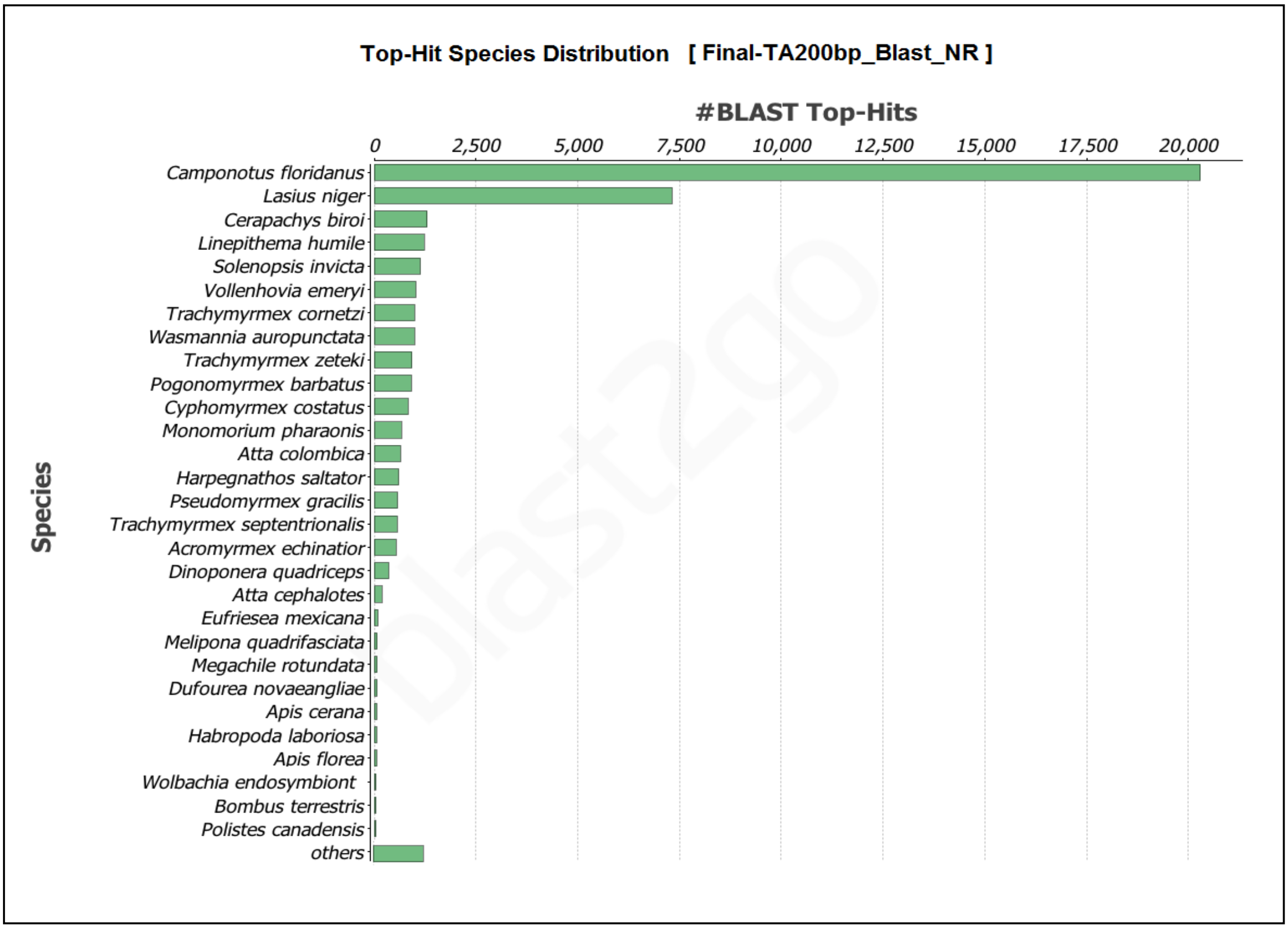

Figure S1-I: Distribution of annotated sequences in different top level GO terms.

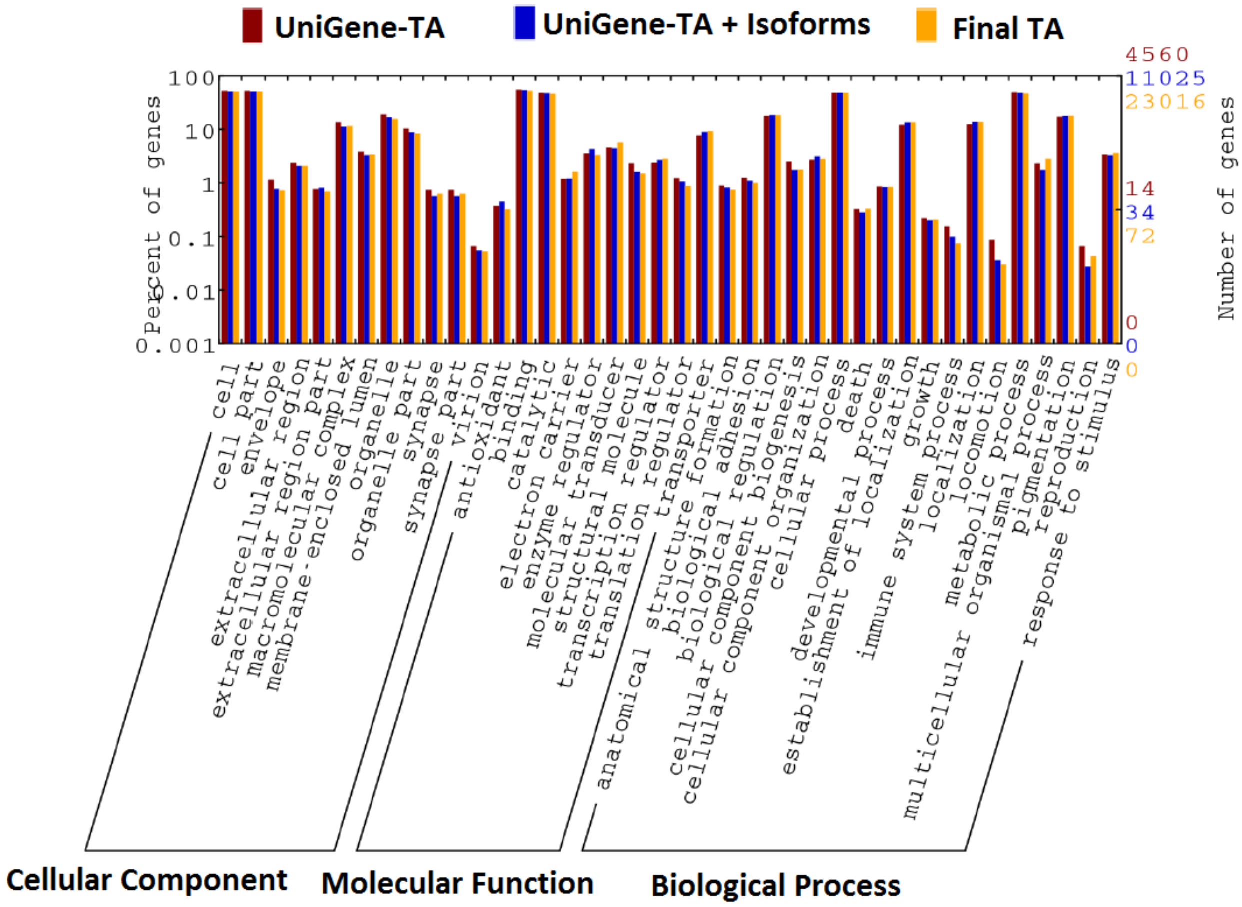

Caste specificity of transcripts in the Final-TA

We assembled caste specific reads (Male, Queen, Worker) separately with the default k-mer (25) of Trinity, and compared that with the Final-TA assembly. Here, we created a similar matrix of coverage distribution of each assembly with the Cflor90 protein dataset to detect caste-specific genes in each assembly.

Table S1-K. Summary statistics of the caste-specific assembly compared to the Final-TA (Mix) assembly.

Assembly stats	Male-TA	Queen-TA	Worker-TA	Final-TA	Badouin et al. TA (Adult Workers)
Contigs generated	75270	105008	120652	118324	30559
Maximum Contig length	12077	26481	32540	28268	9700
Minimum Contig length	101	101	101	200	40
Average Contig length	543.306	641.63	598.825	1039.84	917.282
Total Contig length	40894637	67376272	72249468	123038014	28031212
Total no. of Non-ATGC	0	0	0	0	2723424
Percentage of Non-ATGC	0	0	0	0	0.0971568
Contigs 100 bp	75270	105008	120652	118324	29355
Contigs > 100 bp	45561	59869	66869	118324	28129
Contigs > 500 bp	21609	29092	32376	59151	16205
Contigs > 1 Kb	11459	17770	19362	35367	9296
Contigs > 10 Kb	12	190	74	192	0
Contigs > 100 kb	0	0	0	0	0
N50 value	1165	1780	1634	2041	1543
N80 value	345	418	384	671	566

Table S1-L. Distribution of the caste specific transcripts against Cflor90PP proteins compared to the Final-TA (Mix) assembly.

Cflo90_PP Coverage	Male-TA	Worker-TA	Queen-TA	Final-TA	Badouin et al. TA (Adult Workers)
0-10	171	113	109	86	474
10-20	517	299	308	246	842
20-30	609	366	324	230	696
30-40	642	364	342	230	578
40-50	671	465	395	299	554
50-60	735	649	583	488	618
60-70	664	647	620	567	520
70-80	646	631	616	606	475
80-90	715	778	807	812	479
90-100	4215	5913	6134	7435	2671
Total no. genes covered	9585	10225	10238	10999	7907

Figure S1-J. Visualization of the BUSCO quality metrics. BUSCO was run with four different transcriptome sets (Final-TA, Male-TA, Queen-TA and Queen-TA), and the Hymenoptera reference lineage data sets. Based on the BUSCO assement results, the Final-TA produced a higher number of full length transcripts than the separate caste specific assemblies.

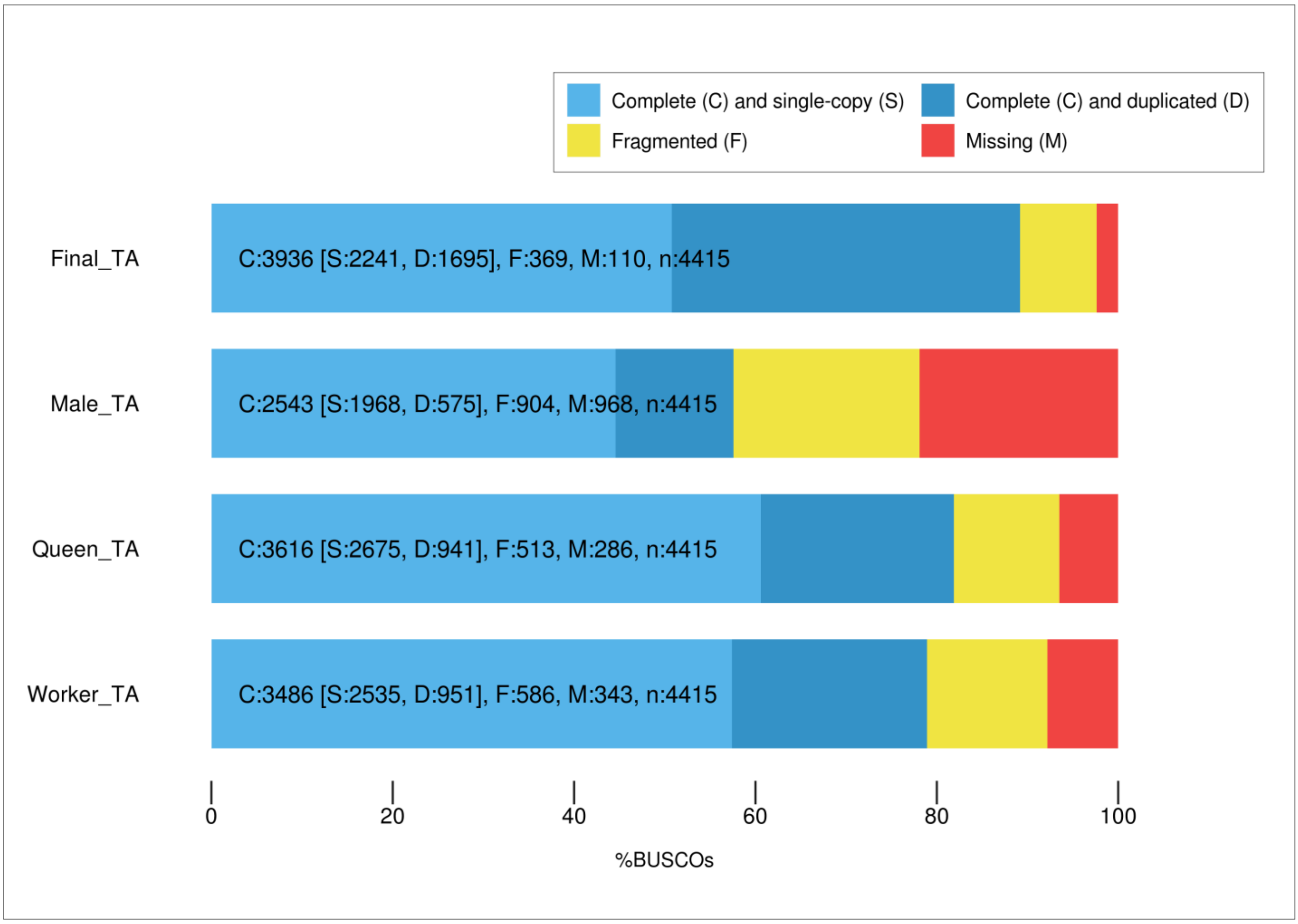

Figure S1-K. Distribution of annotated sequences from caste specific gene sets in different top level GO terms.

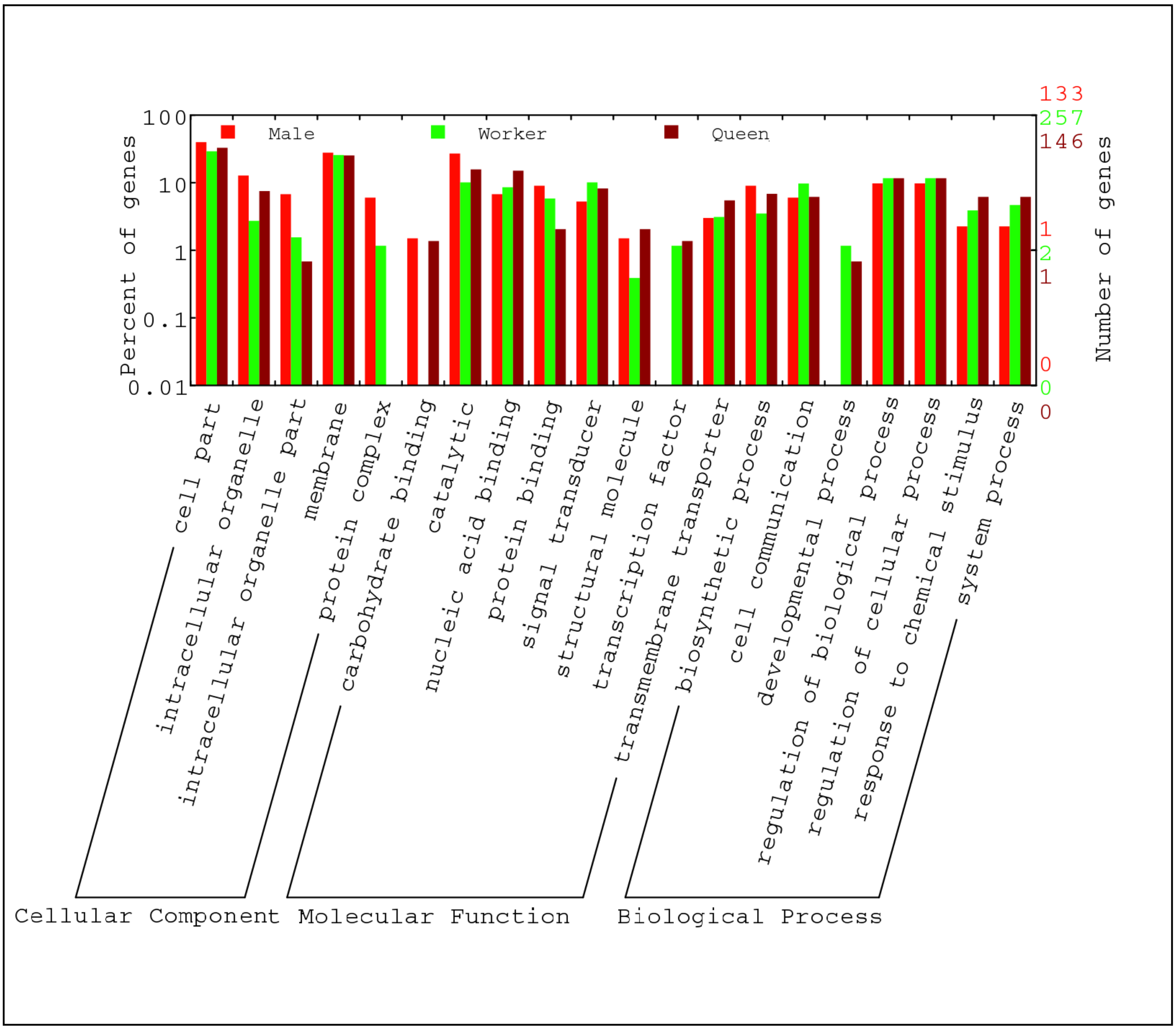

Supplement: File S1 [file peerj-05-3998-s001.pdf]
